# Supplementary material for: Causal interplay between lactose intolerance and gut microbiota: a combined bidirectional Mendelian randomization and in vivo validation study
Source: Front Nutr. 2026 Jun 1;13:1803337. doi: 10.3389/fnut.2026.1803337 (PMC13265576; doi:10.3389/fnut.2026.1803337)
Supplement: Supplementary file 1 [file Data_Sheet_1.zip › supplementary materials/Forward/leave one out cross validation/ebi-a-GCST90027466.finngen_R12_E4_LACTONAS.pdf]

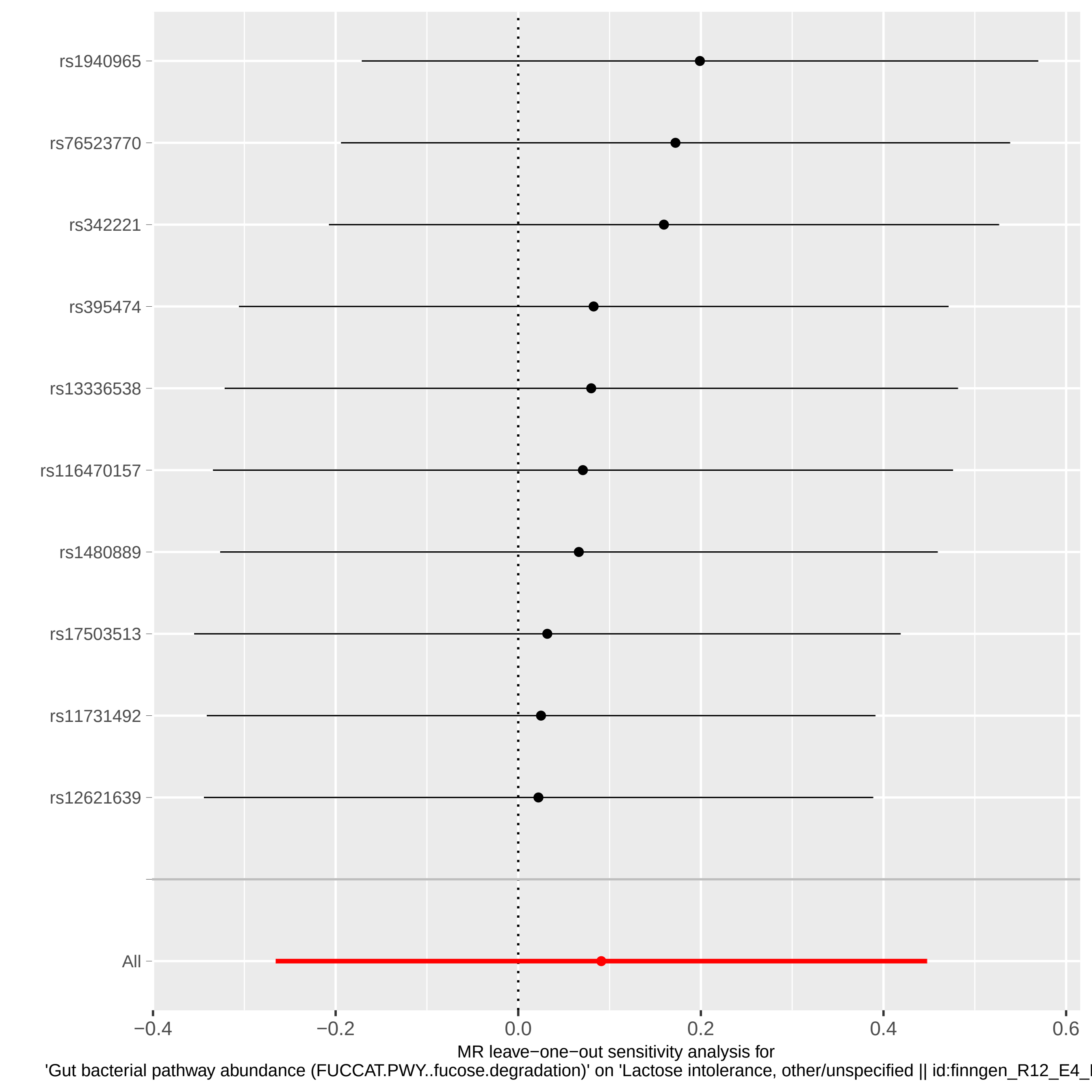
rs1940965
rs76523770
rs342221
rs395474
rs13336538
rs116470157
rs1480889
rs17503513
rs11731492
rs12621639
All
−0.4
−0.2
0.0
0.2
0.4
0.6
MR leave−one−out sensitivity analysis for
'Gut bacterial pathway abundance (FUCCAT.PWY..fucose.degradation)' on 'Lactose intolerance, other/unspecified || id:finngen_R12_E4_
